# Somatic experiencing: using interoception and proprioception as core elements of trauma therapy

**DOI:** 10.3389/fpsyg.2015.00093

**Published:** 2015-02-04

**Authors:** Peter Payne, Peter A. Levine, Mardi A. Crane-Godreau

**Affiliations:** ^1^Department of Microbiology and Immunology, Geisel School of Medicine at DartmouthLebanon, NH, USA; ^2^Foundation for Human EnrichmentBoulder, CO, USA

**Keywords:** trauma, stress, interoception, meditation, somatic experiencing, autonomic nervous system, premotor system, core response network

## Abstract

Here we present a theory of human trauma and chronic stress, based on the practice of Somatic Experiencing^®^ (SE), a form of trauma therapy that emphasizes guiding the client's attention to interoceptive, kinesthetic, and proprioceptive experience. SE™ claims that this style of inner attention, in addition to the use of kinesthetic and interoceptive imagery, can lead to the resolution of symptoms resulting from chronic and traumatic stress. This is accomplished through the completion of thwarted, biologically based, self-protective and defensive responses, and the discharge and regulation of excess autonomic arousal. We present this theory through a composite case study of SE treatment; based on this example, we offer a possible neurophysiological rationale for the mechanisms involved, including a theory of trauma and chronic stress as a functional dysregulation of the complex dynamical system formed by the subcortical autonomic, limbic, motor and arousal systems, which we term the core response network (CRN). We demonstrate how the methods of SE help restore functionality to the CRN, and we emphasize the importance of taking into account the instinctive, bodily based protective reactions when dealing with stress and trauma, as well as the effectiveness of using attention to interoceptive, proprioceptive and kinesthetic sensation as a therapeutic tool. Finally, we point out that SE and similar somatic approaches offer a supplement to cognitive and exposure therapies, and that mechanisms similar to those discussed in the paper may also be involved in the benefits of meditation and other somatic practices.

## Introduction

SE is a novel form of therapy, developed by Levine (1977, 1997, 2010) over the past 45 years. It focuses on resolving the symptoms of chronic stress and post-traumatic stress. SE differs from cognitive therapies in that its major interventional strategy involves bottom-up processing by directing the client's attention to internal sensations, both visceral (interoception) and musculo-skeletal (proprioception and kinesthesis), rather than primarily cognitive or emotional experiences. SE is not a form of exposure therapy; it specifically avoids direct and intense evocation of traumatic memories, instead approaching the charged memories indirectly and very gradually, as well as facilitating the generation of new corrective interoceptive experiences that physically contradict those of overwhelm and helplessness. Why this is an effective approach is the core theme of this paper.

SE shares this focus on internal awareness with traditional methods of meditative movement, such as Yoga, T'ai Chi and Qigong, as well as many forms of seated meditation (Schmalzl et al., 2014). Less well-known Western-grown therapeutic (“Somatic”) systems such as the Alexander Technique (Stuart, 2013), the Feldenkrais method (Feldenkrais, 2005), and Continuum (Conrad-Da'oud and Hunt, 2007), also use this general approach. The explanations and suggestions in this paper apply to some extent to all of these systems.

We believe that the sophisticated and precise theories and techniques of SE offer a way of understanding the processes that occur during mindfulness meditation, both the beneficial mental, emotional and physiological effects of mindfulness meditation and the flooding or dissociation that can occur when traumatic memories surface. In addition, SE can suggest ways in which mindfulness meditation practices could be modified to enable meditators to process traumatic material, and traumatized people to use mindfulness-based techniques to help them recover. At the end of the paper we will elaborate on these ideas.

Over the past 15 years there has been a rapid increase in research on interoception, its relation to the insular and anterior cingulate cortices, and its relevance to the sense of self, cognition, and psychiatric disorders. Craig (2002) and Critchley et al. (2004) have both clarified the efferent and afferent pathways linking the organs to the cortex; Damasio (2003) and Craig (2010) have each suggested a link between sense of self and interoceptive awareness; Damasio, in his theory of somatic markers (Damasio et al., 1996), has suggested interoception is involved in cognition and decision-making. Clear links have been found between compromised interoceptive function and psychiatric disorders, including depression (Avery et al., 2013), anxiety (Paulus and Stein, 2010) and addiction (May et al., 2014). Mindfulness meditation practices have been shown to improve insular functioning and connectivity (Holzel et al., 2011) and to increase interoception (Farb et al., 2013), and insular function has been linked with increased empathy (Singer et al., 2009). Very little research has as yet explored the therapeutic utility of attending to interoception; however see MacDonald (2007) and Price et al. (2007, 2012a). At this point we are not aware of any published peer reviewed studies of SE, neither case studies, clinical trials, nor tests of its mechanisms. While a number of studies are currently underway, more research into SE and its methods and mechanisms are needed. We hope the present paper will demonstrate the possibilities involved in active and structured attention to interoceptive and proprioceptive experience.

We will present a case study of the treatment of a client by SE; this is a composite case, with illustrative episodes drawn from several different cases in the authors' files. The first-person perspective used for convenience during the narrative, also reflects a composite practitioner. We are using this composite case format as a way of succinctly presenting and illustrating the core ideas of SE. Although the interactions are derived from actual clinical experience, bias could be present in the authors' selection of which examples to include. We do not present the case study as constituting evidence for any hypotheses, either concerning SE or other neurophysiological theories discussed.

After each case episode, we will discuss our perspective on the neurophysiology of the events and interventions. The case we present is of post-traumatic stress and pain symptoms following a car accident in which the client was not physically injured but came very close to being killed. This is an example of a relatively uncomplicated kind of trauma: an isolated event, happening to an adult, with no significant complex relational or developmental issues involved and no significant physical damage to the body or brain.

## Case history

The following information is from an extensive pre-session questionnaire Simon was asked to complete before his first meeting with me: Simon is 43 years old man, married with two adult children; he is a middle-level manager at a supermarket chain, normally a competent and well-organized man. Four months ago he was in a car accident: he was driving home from work in the late afternoon at 75 mph on an Interstate highway when a tractor trailer went out of control just ahead of him, colliding with several other cars. He was convinced that he was going to die; but after sideswiping a couple of cars he ended up in the breakdown lane. Apart from a few minor bruises he reported being unhurt; his air-bag went off and he was wearing his seat belt. He was, however, taken to a local emergency room for an examination.

On arriving home that evening, he felt very shaken and teary, but pushed away the impulse to cry and told himself that he should “pull himself together.” The next morning he woke up feeling depressed and anxious, and was unable to organize himself to rent a car and get to work. He became angry with himself. The following day he managed to rent a car and as he began driving to work, he had a panic attack before getting onto the Interstate. He was able to get to work by the back roads, but found himself unable to concentrate at work.

Over the following 4 months he continued to feel “not himself”; he alternated periods of depression and anxiety with bouts of extreme irritability and outbursts of anger, all of which had a negative impact on his work and his marriage. He describes having chronically cold hands and feet, a pounding heart, a knot in his stomach and a fuzzy feeling in his head. Also he notes that whenever he is outside, he has a tendency to be hyper-focused on passing traffic to the point of being distracted from what he is doing. After 2 months, at his wife's urging, he went to see a therapist, but got extremely angry at what he described as the therapist's implication that it was “all in his head.” He says that he knows he should not be reacting this way, that it is not rational, that after all “nothing really happened to him,” but feels completely powerless to change how he feels. Through a friend he heard about Somatic Experiencing, and on being assured it was “not talk therapy,” he decided to give it a try.

## Definitions and terminology

### Autonomic nervous system

When discussing the autonomic nervous system (ANS), pioneering researcher and Nobel prize winner in physiology and medicine, Hess (1925) as well as early researcher Gellhorn (1970) used the terms “ergotropic” (energy seeking) and “trophotropic” (nutrition seeking) to point out that the two principal branches of the ANS cannot be isolated from the somatic and central nervous systems and the neuroendocrine system. The ergotropic system includes activation of the sympathetic nervous system as well as the motor and premotor system (increased muscle tension and preparedness to act), the endocrine system (increased secretions of a number of stress hormones), and the central nervous system (increased sensory alertness), in a coordinated preparation for strong energy expenditure (“fight or flight”). In contrast, the trophotropic system involves these same systems in a preparation for rest, feeding and recuperation. This recognition of an integrated response of the whole nervous system, especially the integration of the autonomic and somatic systems, is central to our thesis.

### The “core response network” (CRN)

**Figure 1:**
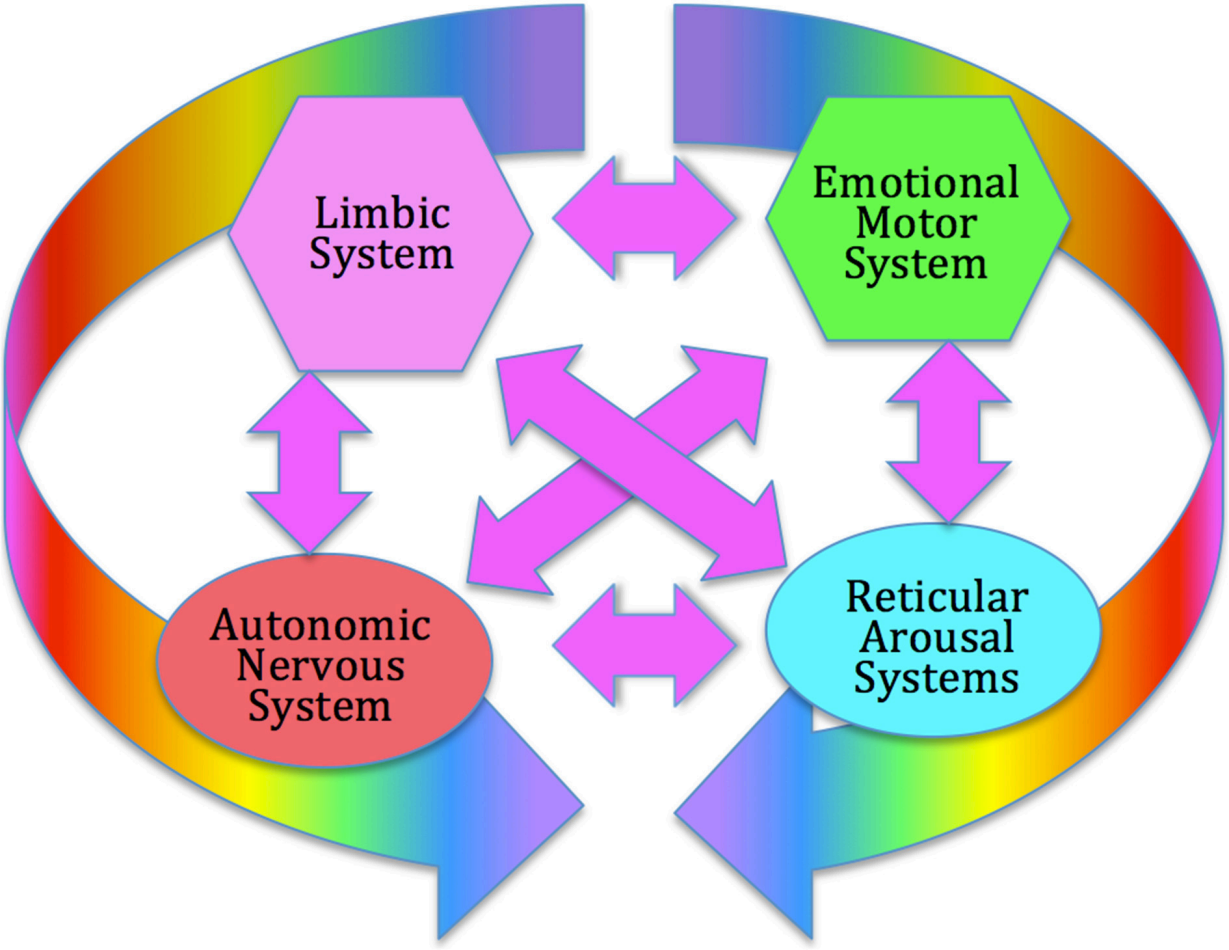
**The Core Response Network (CRN)**. The CRN organizes immediate, instinctive response to environmental challenges, prior to extensive cortical processing. It includes the autonomic nervous system (hypothalamus), the limbic emotional system (amygdala, hippocampus, septal region), the emotional motor system (portions of the basal ganglia, red nucleus, periaqueductal gray), and the reticular arousal systems. All these systems interact strongly through multiple feed-back and feed-forward connections, forming a complex dynamical system which can enter various discrete functional and dysfunctional states.

Unlike conventional psychotherapy which focuses largely on verbal cognitive processes, the focus of SE is on the functioning of the deeper, regulatory, levels of the nervous system, in particular the autonomic nervous system (ANS); the emotional motor system (EMS) (Holstege et al., 1996); the reticular arousal systems (RAS) (Krout et al., 2002; Strominger et al., 2012); and the limbic system (LS) (Heimer and Van Hoesen, 2006); these four subcortical structures form what we term the core response network; see Figure 1.

There is extensive evidence that these four networks interact strongly (Gellhorn, 1970; Weinberg and Hunt, 1976; Hamm et al., 2003; Critchley, 2005, 2013; Thompson, 2005; Coombes et al., 2006; Hajcak et al., 2007; Sze et al., 2010; Kim et al., 2011; Herbert and Pollatos, 2012; Price et al., 2012b; Norman et al., 2014). The ANS can intensify or calm the activity of the viscera, alter blood circulation, trigger hormonal and endocrine activity, change muscle tone, increase or decrease cognitive arousal, and contribute to emotional experience (Norman et al., 2014).

The LS, including amygdala, hippocampus, and septal regions, is central to fear- and pleasure-based experience and to the recall of emotional significance (Heimer and Van Hoesen, 2006). This network has strong bi-directional links to the ANS (Uylings et al., 1999), and the RAS (Strominger et al., 2012), and triggers emotion-specific movement and posture via the EMS (De Gelder, 2006). The RAS involves multiple networks which trigger arousal through several different pathways. It controls alertness and orientation in different contexts, and interfaces strongly with LS, ANS and EMS (Krout et al., 2002; Berntson and Cacioppo, 2007). The EMS involves multiple subcortical motor centers [striatum, red nucleus, periaqueductal gray (PAG)] which are involved in emotion-specific movements and postures which can occur outside voluntary cortical control. It is primarily extra-pyramidal. It is strongly influenced by ANS, LS and RAS, and provides important kinesthetic and proprioceptive feedback to them (Holstege et al., 1996; Holstege, 2013). The CRN responds very quickly to arousing or threatening stimuli, with little input from higher cortical evaluative processes (Porges' “neuroception” Porges, 2004).

This view is very similar to Panksepp's concept of the core self (Panksepp, 1998): a network of largely subcortical structures, centered on the PAG, which are responsible for primal affective experiences and their concomitant motor response organization. We also note the similarity to Damasio's concept of the “proto-self” (Damasio, 2003) and Schore's “implicit self” (Schore, 2011). SE views this core system as the primary target for the treatment of stress and trauma.

### Cortical areas involved in SE

**Figure 2:**
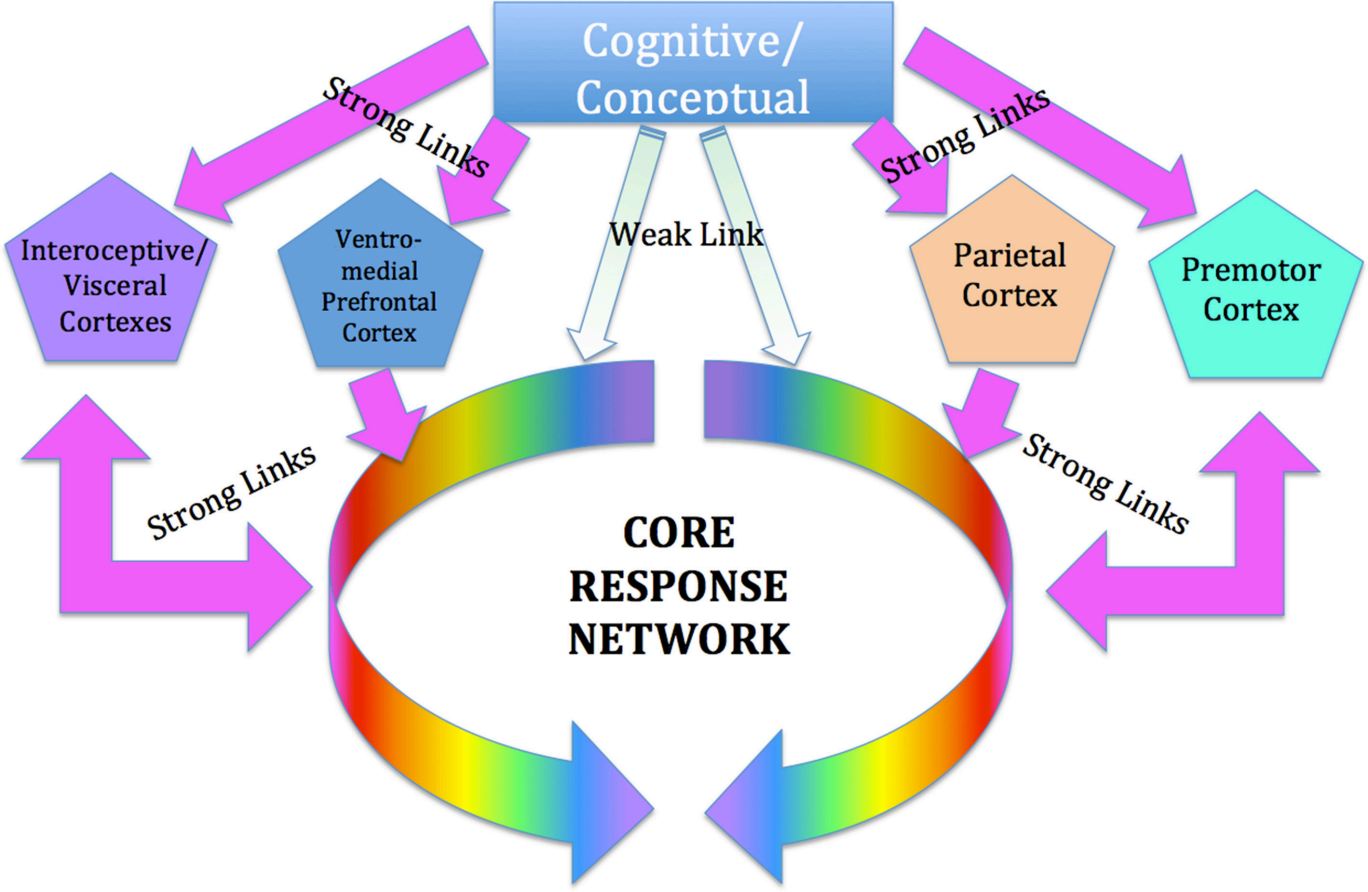
**Cortical control of the CRN**. We suggest that the influence of conscious conceptual thought processes on the CRN is relatively weak and indirect, whereas the influence of those portions of the cortex mediating interoceptive, proprioceptive and kinesthetic awareness is relatively strong and direct. These areas include the insula and anterior cingulate cortex, which have been hypothesized to be involved in cortical control of the ANS; and the sensorimotor and (especially) pre-motor cortex, involved in kinesthetic and proprioceptive experience and in planning and imagining movement, as well as the parietal cortex involved in body schema, and the ventro-medial prefrontal cortex.

We suggest that SE works by restoring optimal function to this network by way of the interoceptive (insula/anterior cingulate) and premotor cortices (Critchley et al., 2003; Craig, 2009). Although words are used in the process of SE therapy, they are used to point to and elicit non-verbal experiences of internal bodily sensation (interoception), sense of position and orientation (proprioception), sensations of movement (kinesthesis), and spatial sense. These are mediated respectively by the insular and anterior cingulate gyrus (Critchley et al., 2003), the premotor cortex (Desmurget and Sirigu, 2009), the parietal cortex (Bartolomeo, 2006; Briscoe, 2009), as well as by the orbitofrontal cortex (Roy et al., 2012). All these areas have very rich and direct communication with the subcortical networks mentioned above, and SE views them as the basis for voluntary intervention on the dysregulated subcortical networks; see Figure 2.

### Stress

Since its first use in physiology, the word “stress” has been subject to multiple definitions and interpretations and the word is often used imprecisely. Hans Selye acknowledged his poor command of English as responsible for a use at odds with that of physics, where “stress” refers to the force acting on an object and “strain” to the resulting distortion; Selye used the word to refer to the response of the organism, and the word “stressor” came to be used for the impacting situation (Rosch, 1986). Stressors may broadly be divided into biological, where the stressor has an unambiguous physical and physiological effect on the organism; and psycho-social, where the effect of the stressor is determined by the interpretation the organism makes of the external situation (Everly and Lating, 2013). Using the same word “stress” to describe the organism's response to these very different categories of events is justified by Walter Cannon's concept of the “stress response” (Cannon, 1970), a supposedly unitary response of the organism to any stressor regardless of its nature.

This early approach led to several difficulties, which have been pointed out by many authors (Levine, 1977, 1986; Lupien et al., 2006; Berntson and Cacioppo, 2007; McEwen and Wingfield, 2010; McVicar, 2013): first, although certain psycho-social situations may be referred to as “stressors,” the event can only be so defined in relation to the response of a specific organism, rendering the definition meaningless (it no longer makes sense to assert that a certain situation “is a stressor” in any absolute or generalized sense). Second, the division into physical and psycho-social stressors neglects the fact that the general state of the organism influences its response to every kind of event, not merely psycho-social events (Vosselman et al., 2014). Some individuals have conclusively demonstrated voluntary (Kox, 2012) and teachable (Kox et al., 2014) control over functions usually believed to be purely “physiological,” such as sympathetic thermogenesis and inflammatory immune responses. The division into physiological and psycho-social is a legacy of the now outmoded Cartesian mind-body separation. Third, current research demonstrates that even the response of the autonomic nervous system to simple physical stressors (pain, temperature, thirst…) is extremely nuanced and individually variable (Saper, 2002), and cannot be summed up as unitary “stress response.” In an effort to resolve these issues, attempts were made to define “good stress” and “bad stress” (Selye, 1975), adding awkward and unwieldy concepts to the mix (Levine, 1986).

Although current views of stress emphasize the role of cognitive appraisal of the stress-inducing situation, recent writers (Porges, 2004; Cohen, 2014) have pointed out that emotionally charged and sudden situations are responded to very rapidly at a sub-cortical level, involving the amygdalar complex and the hippocampus, and not initially engaging the complex associative cortex with its capacity for reasoned decision. In fact much psychological research (Bargh and Chartrand, 1999; Chaiken and Trope, 1999; Cohen, 2014) demonstrates that even apparently rational thought processes are strongly influenced by emotional states. Conscious thought and unconscious emotional processes influence each other reciprocally, it is not a one-way street. Emotional processes equally influence the physical state at the pre-motor level; reciprocally, the state of the body frames the emotional response.

**Figure 3:**
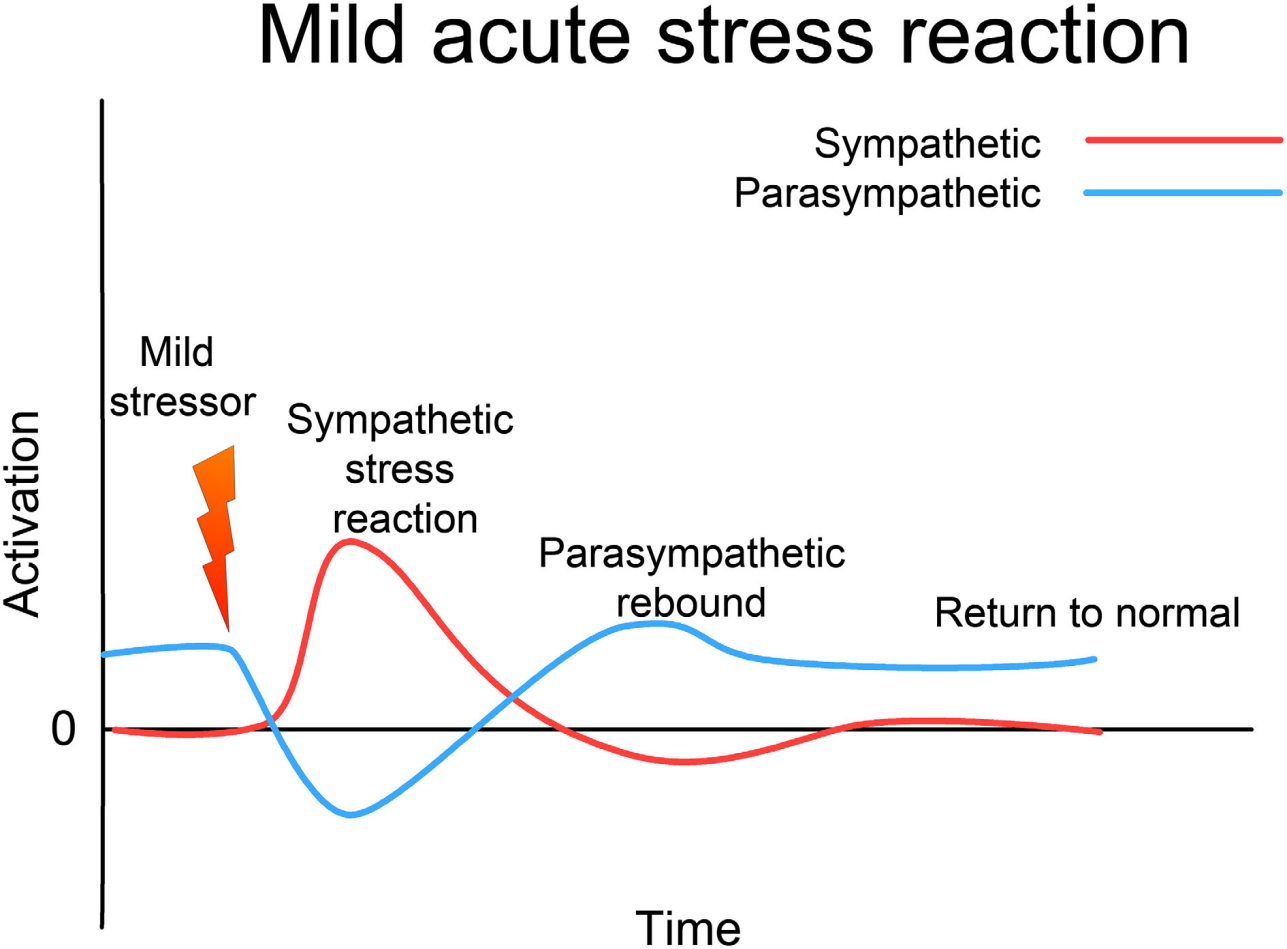
**Acute (mild) stress response**. In response to a mild stressor the ANS (and the whole CRN) responds with sympathetic activation, accompanied by a reciprocal lessening of vagal (parasympathetic) tone. Usually this activation will support an appropriate response to the stressor; this response will be accompanied by proprioceptive feedback that the response has been successfully completed. Sympathetic activation then diminishes, vagal tone returns to normal, and the whole CRN resets to normal resilient functioning.

**Figure 4:**
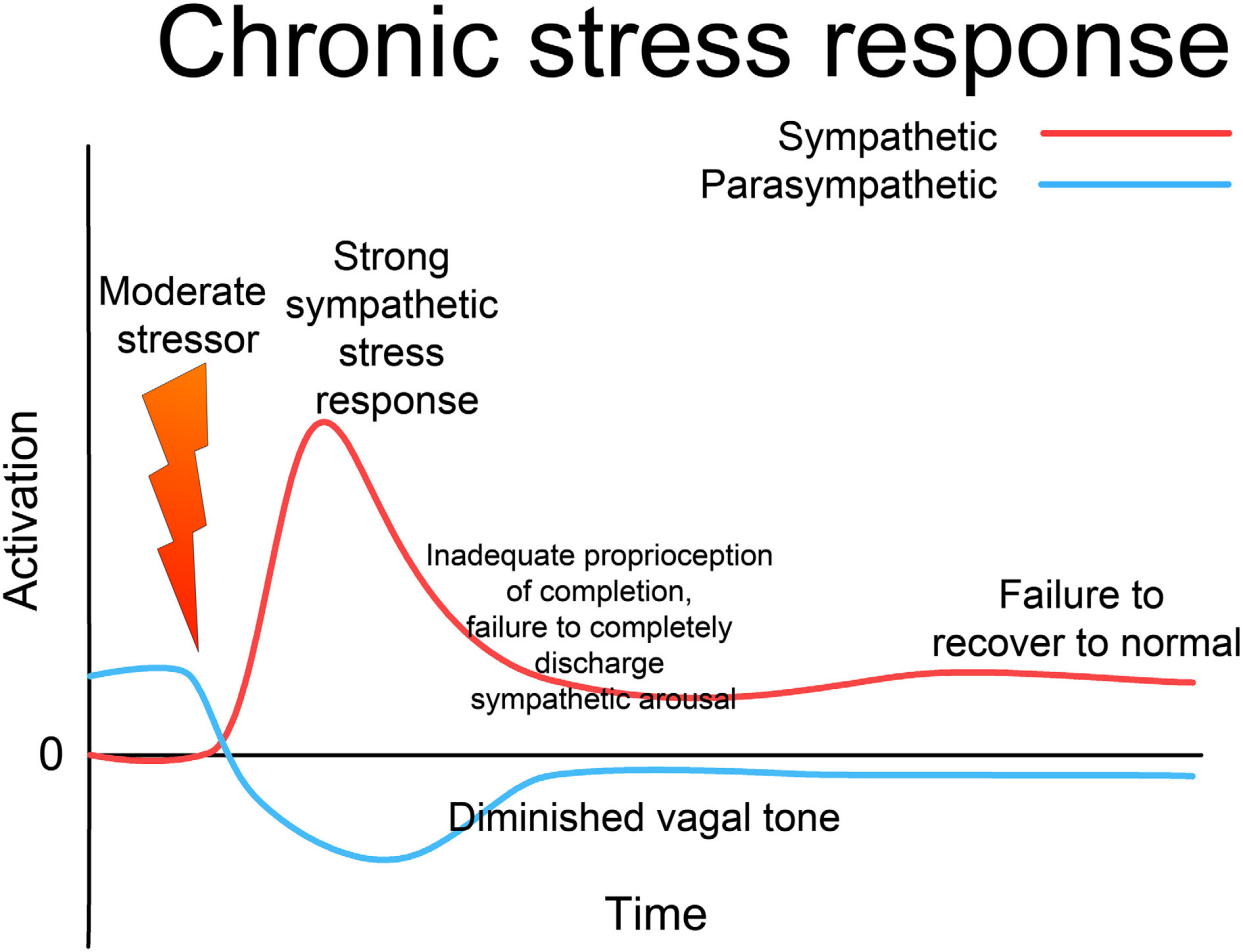
**Chronic stress response**. If the stressor is above a certain intensity or duration, the sympathetic response is more intense; if there is an inadequate defensive response, the system as a whole may fail to reset to normal functioning, remaining “tuned” to excess sympathetic and deficient parasympathetic activation. This state may persist indefinitely, giving rise to a state of “chronic stress,” where the system responds inappropriately to environmental challenge with excess activation. Note that this is not “allostatic wear and tear,” but an altered (dys-)functional state; such a chronic state is a major contributor to allostatic over-load. Through appropriate intervention, the system can be returned to a normalized, fully functional state; but without such intervention the dysfunctional state may last indefinitely.

Since the 1920s, ideas about the functioning of the ANS have evolved from a simple homeostatic linear reciprocal system (Cannon, 1929; Selye, 1954), through concepts of homeodynamics and allostasis (McEwen and Wingfield, 2003; Berntson and Cacioppo, 2007) to the current framework of an allodynamic system, capable of very complex self-regulatory behavior involving feed-back and feed-forward loops and integration with rostral brain centers (Berntson and Cacioppo, 2007). Predating many of these developments, Levine, in his 1977 Ph.D. thesis (Levine, 1977), suggests that the ANS (and related subcortical structures) form a *complex dynamical system* (CDS) (Abraham et al., 1990, 1992). He acknowledges Gellhorn's seminal discovery that, although under normal circumstances the sympathetic and parasympathetic (or ergotropic and trophotropic) systems maintain a reciprocal relationship and return to baseline after disturbance (see Figure 3) following even moderately intense disturbance they can become “tuned” (Gellhorn, 1967a), chronically biased in one direction, and can fail to return to baseline; see Figure 4. In Gellhorn's experiments, rats subjected to stressful stimuli below a certain threshold demonstrated temporary elevation in sympathetic activation and diminished parasympathetic tone, followed by a spontaneous return to baseline levels; however if the stimulus exceeded a certain level of intensity or duration, the ANS did not return to baseline and the rats remained in a chronic state of elevated sympathetic and depressed parasympathetic activity (Gellhorn, 1967a).

Under extreme and inescapable stress, the ANS may start to respond in paradoxical ways, and even manifest simultaneous extreme activation of both sympathetic and parasympathetic branches (Gellhorn, 1964a, 1968). Working with anesthetized cats, Gellhorn clamped the trachea, inducing suffocation. There was an initial extreme rise in sympathetic arousal, followed by an even greater co-activation of the parasympathetic system. This phenomenon has been verified by other researchers (Paton et al., 2006), and is believed to underlie the well-recognized phenomenon of “tonic immobility” (Nijenhuis et al., 1998a; Marx et al., 2008), which is known to occur in both animals and humans under conditions of extreme stress. Gellhorn's animal experiments clearly demonstrate this unexpected behavior of the ANS (Gellhorn, 1970), and Levine clarifies the clinical implications of this phenomenon (Levine, 1977). Levine demonstrates the use of the mathematics of catastrophe theory (Thom, 1989) to explicate and predict the behavior of the ANS under extreme conditions, and relates this model to clinical approaches to treating PTSD and related conditions.

“Stress,” in the sense of an undesirable state, is defined by Levine as the *inability of the complex dynamical system of the ANS to recover to normal functionality* (Levine, 1977, 1986). This is distinct from the current concept of allostatic load in describing stress. Allostatic load refers to the complex neurological and endocrine changes (“wear and tear”) that result from having to make continual adaptations to environmental challenges (McEwen and Wingfield, 2003), but leave the exact nature of the stress response itself still undefined. The “wear and tear” is the *effect* of the stressed condition, and it may lead to circular patterns of perpetuated disruption of normal functioning (Juster et al., 2010). However Levine's approach suggests that to be “stuck” in a “stressed-out” or traumatized state is for the CRN to be stuck in a dysfunctional dynamic mode which is, in principle, fully reversible, and is not determined by the external situation (Levine, 1986). This suggests that (again, in principle) someone whose CRN is fully functional will not accumulate allostatic load in response to challenging environmental circumstances and will thus manifest extraordinary resilience.

### Trauma

As with “stress,” the term “trauma” is used in different ways in different contexts. In SE, a traumatic event is defined as an event that causes a long-term dysregulation in the autonomic and core extrapyramidal nervous system (Levine, 1977, 1997). The implication of this is that trauma is in the nervous system and body, and not in the event; an event that is very traumatic to one person may not be traumatic to another, as people differ very widely in their ability to handle various kinds of challenging situations due to different genetic makeup, early environmental challenges, and specific trauma and attachment histories.

**Figure 5:**
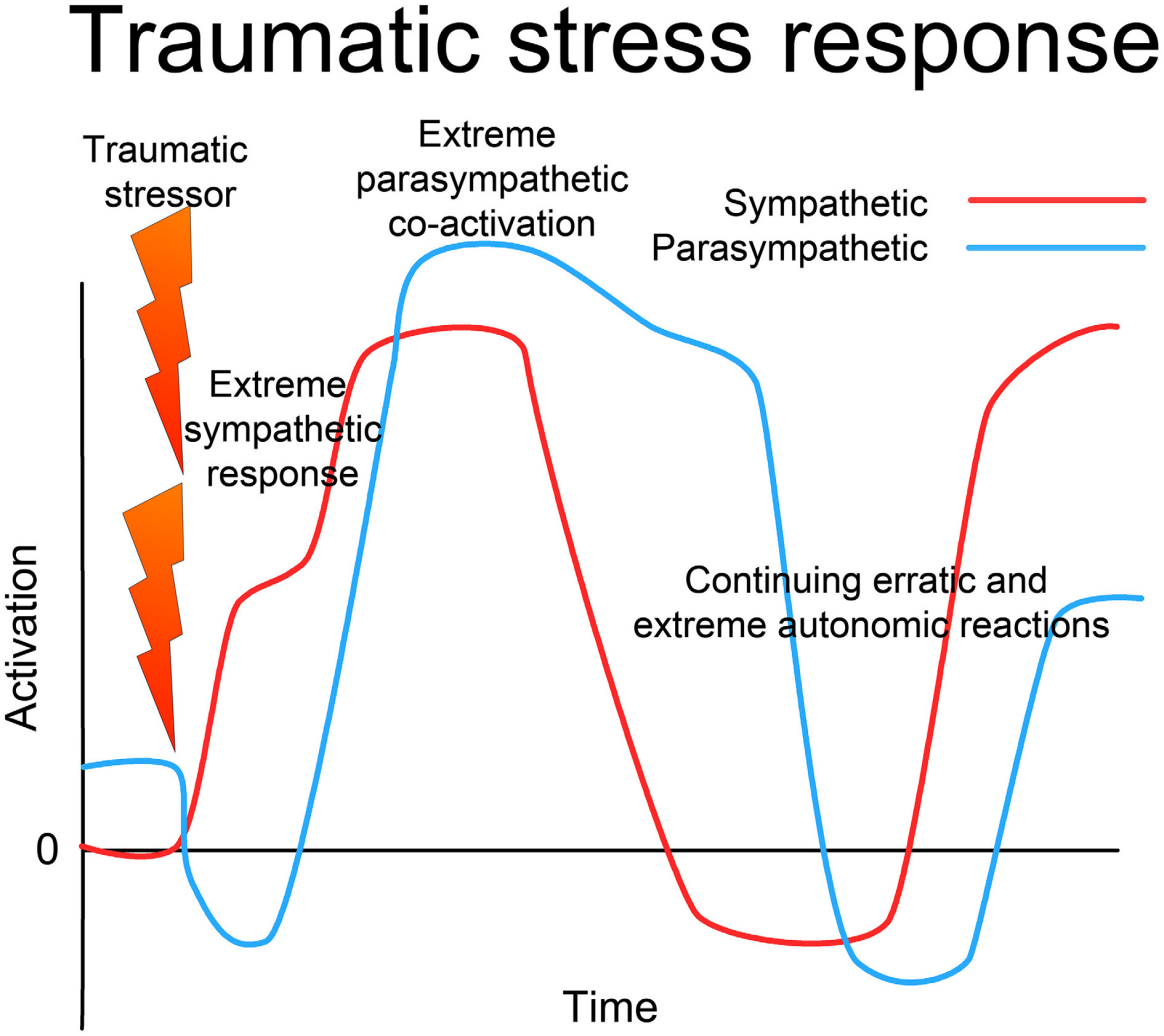
**Traumatic stress response**. In the face of extreme challenge, when either the situation is extremely threatening and overwhelms the capacity of the organism to respond effectively, or if the response is prevented in some way (restraint), there is first an extreme sympathetic (ergotropic) activation with loss of vagal tone. With continued challenge, there is a sudden intense co-activation of the parasympathetic (dorsal vagal) system along with the sympathetic, leading to freeze, collapse or dissociation. The ANS (and whole CRN) becomes locked into a dysfunctional state of extremely high activation of both the sympathetic and parasympathetic systems, and may oscillate erratically between extremes. This may manifest as alternating depressive shutdown and extreme anxiety or rage. This is not the result of wear and tear, but is a specific dysfunctional state of operation of the complex dynamical system, which through appropriate intervention can be returned to normal resilient functioning.

This view implies a continuum of stress conditions; a chronic but mild elevation of sympathetic response at one end, and chronic extreme activation of both sympathetic and parasympathetic (or more exactly, ergotropic and trophotropic) systems at the other. At precisely what point the stress should be regarded as “traumatic” is less important than the understanding of the nature of the dysregulation of the nervous system; however, the phenomenon (demonstrated in cats by Gellhorn, 1964a) of extreme co-activation of sympathetic and parasympathetic systems under life-threatening conditions offers a compelling model for the freeze, collapse, and dissociation often observed in PTSD (Nijenhuis et al., 1998b; Halvorsen, 2014); see Figure 5.

### PTSD

The medical term in common use, post-traumatic stress disorder (PTSD), implies pathology; however SE, (which was developed several years before the definition of PTSD in the DSM III) views the trauma response as part of a natural, non-pathological process that has been interrupted, and therefore prefers the term post-traumatic stress *syndrome* (PTSS) (Levine, 1997). The criteria laid out in DSM IV and V for the diagnosis of PTSD have been challenged by several authors (Shin and Handwerger, 2009; Bovin and Marx, 2011; Scaglione and Lockwood, 2014) and impose limitations not relevant to the theory of SE; most importantly, the DSM V requires exposure to a situation which is threatening to life or body, and limits the range of peri-traumatic emotion acceptable for this diagnosis. Recent authors have pointed to the diversity of various kinds of trauma, suggesting that a unitary diagnosis of PTSD should be replaced by a spectrum of trauma-related disorders (Bovin and Marx, 2011). The theories of SE might provide a framework for such future classification.

## Discussion of these concepts in relation to the case study

Simon, the subject of the SE treatment, was exposed to a situation he perceived as life-threatening, which triggered an emergency (ergotropic) activation response involving the whole CRN: autonomic visceral activation (ANS), immediate terror (LS), great muscular tension (EMS), intense sensory arousal (RAS). That evening his system began a trophotropic/parasympathetic compensation (he felt teary), but he blocked that response. Crying has been recognized as a spontaneous biological activity which can lead to the restoration of balanced autonomic tone (Graèanin, 2014). Cortical appraisal can lead to intentional suppression of emotional behavior or thoughts (Gellhorn, 1969; Wegner et al., 1987; Gold and Wegner, 1995); this has been recognized as a counterproductive, although common, strategy, and involves a (mis-)use of cortical executive networks to interfere in the spontaneous self-regulatory action of the subcortical centers. The central executive network (Szmalec et al., 2005) and the default mode network (Raichle and Snyder, 2007), both involving the dorsal prefrontal cortex, may be involved in this process. These networks are both richly connected to verbal processing areas of the cortex, and exert voluntary control based on held ideas and beliefs (Fogel, 2009); meditation and mindfulness practice have been shown to reduce activity in these networks and instead promote activity in the fronto-parietal network which is engaged in present-centered, interoceptive awareness (Daprati et al., 2010). Conceptually and verbally-mediated control may not take into account the present emotional and physiological needs of the organism. The “mindful” aspects of SE, the gentle encouragement of attention to affective and interoceptive experience, may shift the cortex from dorso-medially to ventro-medially controlled cortical networks (Fogel, 2009) and facilitate spontaneous self-regulation (Herbert and Pollatos, 2012).

Subsequently to Simon's suppression of the tears, his system continued to act as if the emergency situation were still present, and normally neutral stimuli (traffic) took on a new aversive meaning—his CRN remained in an activated state and failed to return to baseline functioning, as a result of cortical executive interference with the re-set process. Although the core emphasis in SE is on restoring subcortical function, it is certainly important to attend to faulty cortical appraisal, and this is best done through methods reminiscent of conventional “cognitive restructuring” (Meichenbaum et al., 2009), verbally addressing the mistaken beliefs and appraisals.

It has been shown that the ANS is subject to both operant and classical conditioning (Grings, 1960; Razran, 1961); a stimulus (passing traffic) which is not inherently aversive may become coupled with one that is highly aversive (an impending accident) such that the former produces the same autonomic reactions as the latter. Simon's description of his physical symptoms (“chronically cold hands and feet, a knot in his stomach”) is consistent with this view. However, unlike conventional or interoceptive exposure therapies (McNally, 2007), SE is not based primarily on a conditioning model, but rather a process model. It has been conclusively demonstrated that autonomic responses are subject to classical conditioning (Razran, 1961), and while we do not doubt that these processes play a role in stress-based dysfunction, the stimulus/response model has long been recognized as inadequate for explaining complex behavior. Control systems, such as the systems involved in autonomic regulation, require feedback and feed-forward loops which are not part of the explanatory framework of conditioning theory (Haken, 1977). Although we do not question the well-established knowledge concerning neuronal dendritic modification in response to conditioning, the behavior of complex neural networks are governed by higher-order principles of dynamical systems theory (Haken, 2012). Thus, in SE, symptoms are seen as due to a disorganized complex dynamical system, rather than resulting from a simple conditioning process (Levine, 1977). Fear conditioning extinction is the canonical model for recovery from PTSD, especially through exposure therapy (Rothbaum and Schwartz, 2002); however conditioning theory states that, in the extinction process, a conditioned fear response is not actually eradicated but only suppressed by competing (positive) conditioned experiences (McNally, 2007); the implication of this, born out by experience, is that, although fear de-conditioning is quick and effective, it is also easily disrupted, as re-exposure to trauma-related cues easily reinstate the fear response (Vervliet et al., 2013). By contrast, clinical experience in SE demonstrates a very robust change in fear responses which are remarkably resistant to re-evocation; this is consistent with the theory that clinical changes mediated by the SE process are not primarily due to fear conditioning extinction but to a discontinuous alteration in CRN dynamical functioning; in terms of dynamical systems theory, a shift to a different attractor basin (Abraham et al., 1990, 1992).

Simon's inability to have volitional control over his reactions is also consistent with the idea that the dysfunctional ANS/CRN is the core issue; the CRN is not normally under the direct control of conscious volition, and is relatively unaffected by rational thought processes (“he knows he should not be reacting this way, that it is not rational, that after all ‘nothing really happened to him,’ but feels completely powerless to change how he feels”; such comments, in our clinical experience, are quite common). This points to a drawback in “talk therapy” for trauma; the SE perspective is that the CRN is most effectively addressed through interoceptive and kinesthetic awareness.

Simon's nervous system is now clearly dysregulated. It is unable to return to baseline, and is oscillating between extremes of activation (ergotropic, anxiety and rage) and shut-down (trophotropic, depression and numbness). From the point of view of SE, this current state of Simon's nervous system is the relevant fact, not the objective nature of the triggering event itself nor even the conscious peri-traumatic experience (Simon's experience at the time of the traumatic event).

## The sessions

Selected portions of the four SE therapy sessions are presented, interspersed with commentary.

### 1st session, 1st half

When Simon first came into the office, his shoulders were elevated, his breathing high in his chest, his tread heavy; his face was frowning, his jaw clamped, his eyes narrowed. I had the impression of a tense, defiant attitude; I imagined he was ready for a confrontation, given his reaction to a prior “talk psychotherapy” session. I greeted him, introduced myself, and offered him his choice of chair—there were several different chairs in the room. He seemed slightly disconcerted at being offered a choice; he paused, looked around the room, took a deep breath, glanced back at me, and settled purposively in the most comfortable-appearing chair. As he shifted in the chair he looked at me again; I imagined he might be wondering if he had taken my chair, and could be feeling a bit defiant in anticipation of my reaction.

Me: Good choice. I think that's the most comfortable, it's for the most important person here: you.

Simon: (looks at me with slight surprise, the frown lessens, he moves in the chair again as if testing its comfort). OK.

Me: (sitting down) How does that feel?

Simon: Yeah, good, it's comfortable, thanks. (He takes a deep breath, closes his eyes for a moment, his shoulders drop, his body appears to relax more into the support of the chair. He opens his eyes again and looks at me; this is the first time he has really looked at me).

Me: (I make brief direct eye contact with him, settling into my own chair) Before we get started, I'd like you to really notice how it feels in your body as you get more comfortable in that chair. What's that like physically?

Simon: (Moves his shoulders a little) Uh, well… I notice it in my shoulders I guess. And my arms, they feel more relaxed. (Frowns slightly as if concentrating.) I feel kind of, like heavy I guess—a good heavy—and warmer. (Heaves a sigh). I feel kind of relieved.

Me: OK good, relieved; and as you feel that, can you notice any other areas of your body that feel, a bit, the same way?

Simon: (Pause, shifts his body a bit, appears to relax further; closes his eyes) My chest feels more relaxed; and I guess my legs feel better too, like they are resting more. (Abruptly opens his eyes, his breathing speeds up a bit, he tenses up a little) Shouldn't we be talking about the accident?

Me: (I make gentle relaxed eye contact) Yes, we will get to that very soon, I do want to hear about it; but first, for what we are doing here, it's really useful for you to notice how relaxed you can get; this will be really helpful. You know, if you are about to climb a big mountain, you don't just head out dressed in a T-shirt; you first get good clothes, boots, a guide—all the things you will need. Well, getting in touch with good feelings in your body is like gathering the things you need to deal with the difficult stuff later. So… just noticing those relaxing feelings… how is that?

Simon: (his voice shifts, becomes more resonant and softer; he moves his jaw slightly as if chewing) Good—actually I feel really good, don't remember when I felt this good since the accident … (pause, sighs;) it's been such a strain… (his voice becomes a little throaty as if he were about to cry, I notice slight tearing in his eyes. I recognize sadness coming up, and I anticipate, based on his pattern of “keeping it together,” that he may quickly tense up against it, so I support this feeling).

Me: (In a soft voice) Yeah, such a strain… I understand… it's OK to feel that, just let yourself feel that, its fine… such a relief to feel a little better…

Simon: Sorry, I don't know why…. (Some more tears, then he relaxes and settles, opens his eyes and looks at me; I meet his gaze then look away, meet then avert, to show him I am present and supportive, but not challenging him to open up more than he already has; I am aware he could easily feel ashamed at me seeing him so vulnerable.)

Me: Yeah… how are you doing now?

Simon: Wow, a lot better, feels like a big load off me. What… is this normal?

Me: (I reassure him and explain some more about the SE process; some of what I tell him is in the discussion below. It is very useful for a client to have a clear understanding of the SE process, as much of it is unlike anything else they may have experienced previously, and is often somewhat counter-intuitive compared with their assumptions about what they need to do to free themselves of trauma).

#### Discussion

The session begins the instant Simon walks through the door. With the knowledge gleaned from the pre-session questionnaire as background, I am immediately observing cues as to the state of his nervous system, and am choosing to act in particular ways on this basis. My initial goal therefore is to bring Simon into a state of safety and comfort, in which his CRN is more balanced. In SE this is known as “*resourcing*”; to put a person in touch with positive inner feelings of safety, strength, comfort, and optimism, so that they can begin to take the steps which can lead to stable restoration of balance. These are not abstract mental states of well-being, but embodied experiences of positive feeling: an important distinction in SE.

One of the principal ways I do this is through social engagement, with the use of eye contact and voice. Porges (2007) postulates that the ANS has three, not two, divisions. While the sympathetic is associated with mobilization in response to threat, the parasympathetic serves to support survival through its two different evolutionary branches, the dorsal and ventral vagal complexes. The evolutionarily older system, the dorsal vagal, promotes shut-down and immobility, while a more recent branch, the ventral vagal, governs social engagement. This includes the supra-diaphragmatic vagus as well as the cranial nerves which serve eye contact, speech, hearing and feeding behavior. Porges suggests that the ventral vagal serves as a complex and nuanced way of inhibiting excess sympathetic activation (“stress”) through engaging socially with others. SE makes considerable use of this system to promote nervous system balance. In addition to eye contact and verbal interaction, I use whatever presents itself as useful for putting him at ease and encouraging positive sensation–in this case his choice of chair, though every situation is different and it could just as well been his glance at a painting on the wall or a certain kind of sigh. Notice that in the description I often use the phrase “I imagine…” when describing my observation of his inner state. This is intentional, and expresses the truth which, as a therapist, I have to continually keep in mind: all I actually see are certain outward behaviors; I then project what these mean in terms of his inner state; but I could always be mistaken. So if I am to have accurate observations, I must remember this and be ready to change my evaluation if it is contradicted.

I am specifically guiding Simon to notice positive inner sensations as they arise. Most people, especially those who are stressed or traumatized, tend to focus immediately on negative interoceptive cues as harbingers of their distress. Damasio refers to interoceptive cues as “somatic markers” (Damasio et al., 1996, 2000), which emerge into consciousness via the insula (the interoceptive sensory cortex), and suggests they have a significant role in contacting one's instinctive or pre-conscious judgments about the environment. By avoiding interoceptive cues one reduces one's capacity to evaluate the environment; by focusing on negative cues only, one increases fear reactions. An important initial step in SE is to draw the client's attention to positive, non-aversive somatic markers; this brings the ANS and subcortical emotional centers into a less fearful state, as well as enhancing the connection of the frontal cortical centers with the subcortical. Critchley (Critchley et al., 2003, 2004; Critchley, 2013) suggests that the insular and anterior cingulate cortices are the top level of control for the ANS, forming a regulatory loop involving interoceptive sensory and motor cortices, amygdala, hypothalamus, and brain stem nuclei; one of SE's effects may be to enhance the functioning of this loop, thus promoting improved functioning of the subcortical centers. This is accomplished by attention to interoception rather than to cognition.

**Figure 6:**
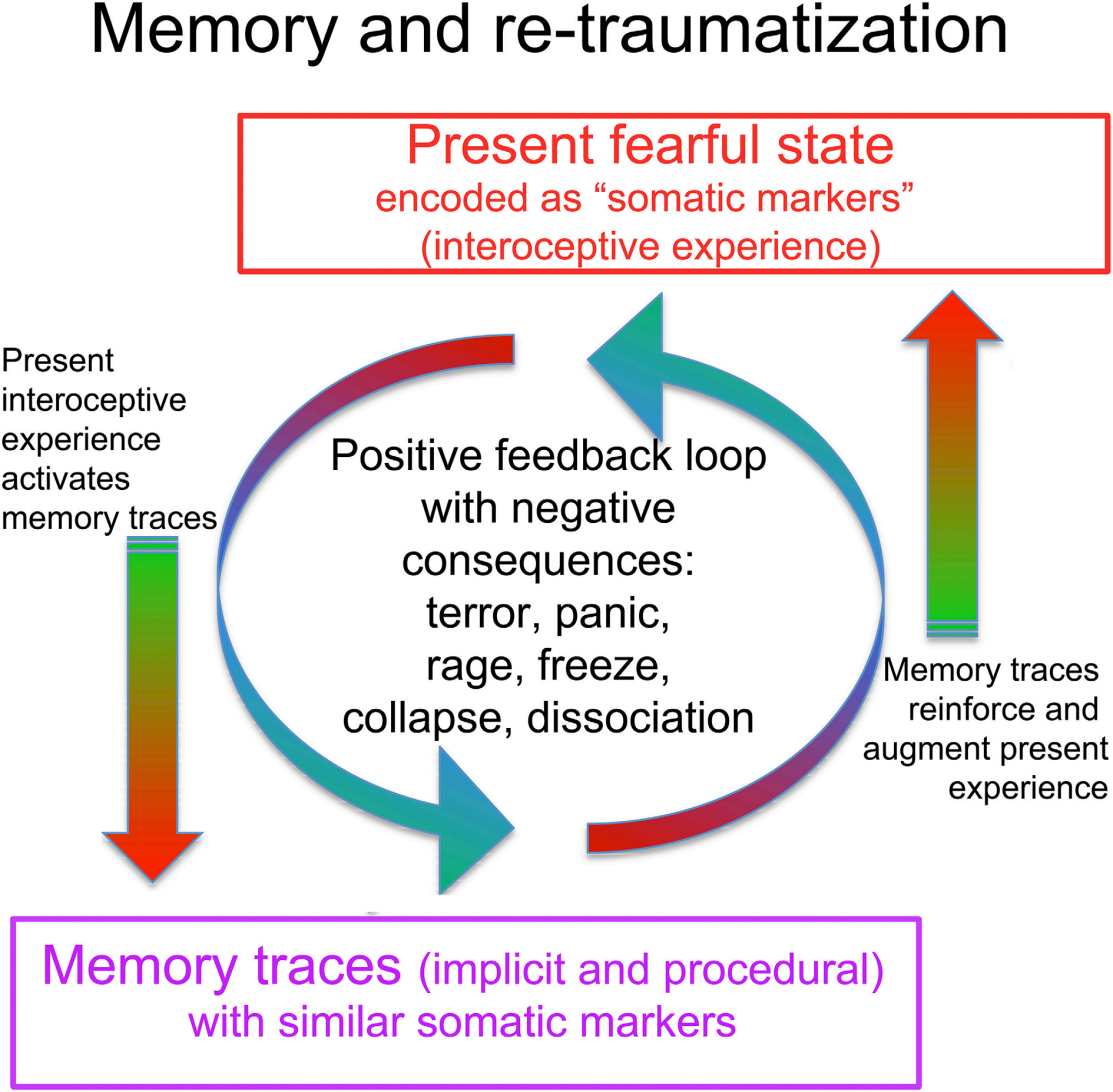
**The interaction of traumatic memory with the present state**. A present fearful or stressful state is experienced in part as unpleasant interoceptive and proprioceptive feelings, including muscle tension, stomach tension, trembling, weakness, constriction, increased blood pressure (pounding pulse), decreased blood pressure (dizziness), increased or decreased heart rate, cold sweaty hands, hyperventilation, shallow breathing. Damasio terms these “somatic markers,” as they are bodily experiences of emotionally and viscerally activated states, consciously felt “markers” of subcortical states. These somatic markers may activate memory traces that contain similar feelings. Such trauma-related memory traces may be partly or wholly inaccessible to ordinary conscious recollection, being procedural or implicit rather than declarative and autobiographical. This means the person may not even be aware that old memories are being activated. Consciously recognized or not, the somatic markers connected to the old memories reinforce and augment the present fearful state in a runaway positive feedback loop, which can lead to terror, panic, rage, or shut-down. In response to these aversive experiences (whether triggered by a present situation, conscious memories, or implicit and procedural traumatic memories), the CRN mobilizes a defensive response; given the circumstances, the response is unlikely to succeed (unless carefully guided by a skilled therapist). Such renewed failure may further disorganize the system and add to the undischarged activation (re-traumatization).

At first, the session description may seem like no more than a relaxation induction. However, at a certain point Simon abruptly shifts direction, tenses up, and brings his attention back to the trauma (“Shouldn't we be talking about the accident?”) This is an example of a phenomenon which can also occur in meditation or other relaxation-oriented therapies: deep relaxation may trigger a sudden upwelling of aversive material (Everly and Lating, 2013); at the end of this paper we briefly suggest that the SE perspective may offer effective ways of dealing with such difficult experiences, enhancing the therapeutic benefit of relaxation- and mindfulness-oriented therapies. If he were to follow this trauma-oriented impulse it would likely rapidly lead to a vicious cycle of intense fear, sympathetic arousal, loss of clarity, intrusion of memories, increased distress, and a state in which further therapeutic progress would be difficult (see Figure 6, below, for an illustration). Yet Simon is correct: the trauma around the accident cannot and should not be avoided indefinitely. My explanation about “resource” makes sense to him and allows him to return for a while to a subjectively pleasant state. This enables a large, spontaneous shift: the reduced sympathetic tone allows a parasympathetic increase, and with some more tears (Graèanin, 2014) comes a gentle sense of relief, an acknowledgment of the strain he has been under. Had we tried to engage memories of the accident full-on, the resultant sympathetic activation might have blocked the possibility of this kind of gentle discharge. As it is, he is left in a significantly more relaxed and functional state, prepared to go a bit deeper in the rest of the session. This going back and forth between charge/activation and discharge/deactivation needs to be finely tuned. Too much of one or the other, and the process of re-establishing balanced functioning is interrupted. This distinguishes SE from exposure therapies, which do not tend to avoid extremes of activation. SE terms this back and forth process “*pendulation.*” When skillfully nurtured it tends to occur spontaneously as the system seeks to restore balance (Levine, 1997, 2010).

Our view is that the subcortical systems (CRN) have intrinsic mechanisms for restoring inner regulation and autonomic balance; it is the role of the SE therapist to facilitate this process. Ongoing cortical executive suppression of behavior (crying, tearing), thoughts or feelings is counterproductive to this spontaneous restorative process (Gellhorn, 1969). By creating a safe environment and gently re-framing Simon's interoceptive and emotional experience, I enable him to withdraw suppressive cortical control and to approach his inner experience in a graduated (titrated) way. This reduces excess sympathetic arousal and consequent suppression of frightening interoceptive experiences, which in turn facilitates the intrinsic regulatory process of autonomic discharge and the restoration of sympathetic-parasympathetic balance. This approach can be contrasted to the more repetitiously confrontative approach of exposure therapy (both conventional and interoceptive) (Rothbaum and Schwartz, 2002; Wald and Taylor, 2008); we believe SE accomplishes fear extinction more quickly and with much less distress, probably via a different mechanism than that postulated for exposure therapies: “biological completion,” as described below.

### 1st session, 2nd half

Me: OK, so let's do something here. So what was the weather like the morning of the incident?

Simon: Oh, the weather? Umm…I guess it was nice, yeah, a nice day. I had no idea…

Me: (interrupting) OK Simon, see if you could just focus on your memory of the weather when you first left the house, before you even looked at the car! What were you doing? Can you remember the sunshine, the temperature…?

Simon: Oh…OK…well, yeah, it was really clear, it was crisp.

Me: (noticing his breathing speed up and a slight trembling in his hands) Hmmm, so, right now, what are you aware of, Simon?

Simon: Well, I feel a little tense I guess…

Me: So it is just a little? Is that OK?

Simon: Yeah, not too bad… I can manage it.

Me: OK good, see if you could just allow that tension, just as it is…what do you notice?

Simon: OK, well, my shoulders are a bit tense…I kind of feel a bit shaky…

Me: OK, see if you can stay with that Simon, that's fine, just notice that little shakiness. Where do you sense that?

Simon: Yea, that's strange, my hands are shaking…

Me: You're doing great Simon, that's good; just stay with your awareness of the shaking…what happens next?

Simon: I feel the shaking spreading up my arms—this is weird–

Me: It's OK, just see if you can be with it Simon, it's just your body releasing tension, just let it happen… (pause)… and what's that like now?

Simon: Oh, I feel shaky all through my chest (voice thickens) I feel a bit teary—what's happening?

Me: You are just letting go of a bit of tension Simon, let it happen (making eye contact).

Simon: (shakes visibly, sighs a few times, closes and opens his eyes. Gradually the shaking subsides) Wow, that was weird!

Me: How are you doing?

Simon: OK I guess, good. (Breathes deeply.) Fine. That was weird!

Me: Simon, when the body gets tense it has natural ways of shedding the tension—sometimes we cry or shake, sometimes we yell or yawn, it's just natural. But we are not used to letting these things happen, so it's unfamiliar…. So—you were telling me about the weather on that morning….

Simon: Oh yeah…well, like I say, it was clear, crisp…I can remember my ears feeling cold, there was a bit of wind….

Me: Do you hear anything?

Simon: Well, the wind sound, the birds—some traffic in the background….

Me: How do you feel in your body as you recall that?

Simon: Fine, I feel relaxed… hey, I just noticed that the sound of the traffic doesn't bother me right now!

#### Discussion

The second half of the first session demonstrates the core of the methodology of SE. The first important concept is that of “*discharge.*” The sympathetic nervous system mobilizes the body for intense kinetic activity (“fight or flight”). Under normal circumstances this “biological energy” (the secretion of various neuroendocrine substances and activation of certain neural pathways) is used to power intense muscular activity; when successful, this arousal is part of a cycle involving mobilization, successful action, exhilaration, relaxation, and a return of the nervous system to baseline functioning. However, under certain conditions the ANS may get “stuck” in a state of excess activation; the muscular activity does not happen or is not successful, the reciprocal activation of the parasympathetic is not triggered by proprioceptive feedback, and the system does not return to balance but continues to secrete activating neuroendocrine hormones (Gellhorn, 1969). Gellhorn has clarified that the proprioceptive feedback from intense muscular activity is the trigger for the reciprocal activation of the parasympathetic (Gellhorn, 1964b). Rats allowed to fight with each other after a stress-inducing experience recover much more quickly than rats kept separate and thus unable to fight (Weinberg et al., 1980). Even in the absence of this trigger, the nervous system nevertheless has ways it can release the excess activation; this usually involves spontaneous movement of the body (including gentle shaking and subtle postural changes), often accompanied by feelings of fear, sadness, or relief (Levine, 2010). Drawing the client's attention to the proprioceptive and kinesthetic (somatic) markers of this “release” process serves to enable a spontaneous re-balancing of the nervous system. We have already discussed crying above; shaking and trembling are very little referred to in the literature. There is slight mention of trembling as a component of what has been called “rape-induced paralysis” (Galliano et al., 1993), which is believed to be closely related to “tonic immobility” (TI), an innate biological reaction to extreme stress (Marx et al., 2008; Volchan et al., 2011). From an SE point of view, this trembling or shivering is an opportunity for therapeutic intervention; it is a sign of the system's attempt to begin restoring normal function. Shivering is triggered in the pre-optic area and is associated with thermogenesis (Nakamura and Morrison, 2011). It helps maintain optimal conditions for muscle function in preparation for vigorous defensive activity. We speculate that the trembling observed in TI may be a preparatory sympathetic reaction attempting to warm the muscles in preparation for a defensive response. Encouraging this physiological process could lead to vigorous sympathetic activation, the expression of blocked defensive reactions, and the facilitation of a parasympathetic rebound to normal ANS function. An SE therapist would reassure the client that the shivering is a natural process and encourage the movement to develop into a possibly empowering response.

The second significant concept illustrated is *titration*. This term is used in chemistry to describe the process where two reagents (like a strong acid and strong base) are mixed drop by drop to avoid the explosive reaction that would occur from pouring them together quickly. It is also used to describe a process of carefully and slowly introducing a new drug to determine the correct dosage for an individual. In the same way, trauma must be approached very slowly, “drop by drop,” so as to avoid unnecessary distress, flooding and potential re-traumatization. Note the care with which I prevent Simon from following his inclination to go straight to thoughts of the accident, and how we instead begin by attending to experiences far removed from the trauma itself. Even these bring up some degree of activation, but at an easily manageable level, such that discharge can occur without undue distress. Once a little discharge has happened, the ANS/CRN is in a somewhat more balanced state, and Simon can then tolerate more discomfort of arousal, discharge and further regulation and resilience in the next go-round.

I anticipate that Simon might experience some re-activation of the trauma during the coming week, but my expectation is that a significant amount of the pressure has been let off, so he is unlikely to experience a lot of distress, and I think he will return next week with a more resilient system and well prepared for deeper work.

### 2nd session (partial)

Simon enters my office looking noticeably happier than last time. His posture is more upright and he is smiling. He greets me warmly, we shake hands, he sits again in the same seat. We make brief direct eye contact.

Me: So, how's it going?

Simon: On the way home I got a little freaked out by the highway again, but I knew it was going to be OK. But, I certainly felt a lot better.

Me: Alright, that makes sense; tell me, what were the good feelings like after the session?

Simon: Oh, I felt really relaxed, all that tension dropped away; it felt like such a relief. (He sighs and settles into the chair)

Me: And what are you noticing in your body while we are sitting here talking right now?

Simon: I feel good—must be this chair! (Smiles mischievously and laughs).

Me: So… let's come back to that morning, remembering how that was…what do you notice happening in your body as you recall that morning?

Simon: I feel fine, no problem, I can remember that scene fine.

Me: So, where was the car? (At this point I observe Simon carefully for the first signs of activation; I want to elicit some activation to work with, but not so much as to lead down the slippery slope toward overwhelm).

Simon: (calmly) In the garage.

Me: OK, so, do you remember how you got to it?

Simon: Yes, I went and lifted the garage door.

Me: OK, simply remember doing that, and notice how you feel as you explore that image.

Simon: (still appearing relaxed) Well, I see myself opening the garage door…I am going to the car door…I am getting in…

Me: (noticing Simon's shoulders come up, his breathing getting more rapid) OK, let's pause for a moment. What do you notice?

Simon: (suddenly closing his eyes, sitting forwards in the chair, twisting his body a bit to the left, hunching his head down; his voice sounds tight) Oh Jesus that was so scary, I really thought I was going to die!

Me: (firmly) OK Simon, slowly begin to open your eyes…Simon, look at me, right here. (Simon slowly opens his eyes, at first he looks at me vacantly, his breath rapid) You're fine Simon, you are right here, it's OK. Just see me, right here. (Simon's eyes come back into focus, his breath slows).

Simon: Oh damn, what happened?

Me: (in a calm voice) It's fine, we just went a bit too quickly. Look around the room a bit, tell me three things that you see.

Simon: (focusing on the room, his voice calmer and slower) OK…I see the walls…your picture there…the window…

Me: Can you feel the chair?

Simon: Yes—the magic chair! (Chuckles) That's better!

#### Discussion

Despite my attempt to keep things slow, Simon slipped into the “trauma vortex”; the memory of getting into the car triggered an intense recollection of the accident accompanied by strong activation of the ANS and the rest of the CRN, and I had to act quickly to bring him back to the present so that his nervous system could regain its balance. In SE one is walking the tightrope between not enough activation, in which case there is no discharge because there is no activation to discharge; and full-blown reactivation of the trauma memory, in which aspects of the trauma are relived and the person again experiences overwhelm. This can actually be harmful, and can compound the original trauma. Such a “dive” into the black hole, the “vortex of trauma,” involves a self-reinforcing positive feedback loop, in which the proprioceptive and interoceptive feedback (somatic markers Damasio et al., 1991, 1996) from the neurally encoded memory trace (engram), becomes a trigger for further activation (Liu et al., 2012); a runaway loop which can lead to extreme simultaneous activation of both sympathetic and parasympathetic (dorsal vagal) bringing about a dissociated state within seconds; see Figure 6. One of the tasks of SE is to interrupt this destructive loop. To this end, SE uses concurrent evocation of positive interoceptive experiences, which may help alter the valence of the disturbing memories (Quirin et al., 2011); this process has been demonstrated in rats (Redondo, 2014). Other aspects of the mechanism whereby SE prevents the traumatic positive feedback loop are discussed below as “biological completion.”

### 3rd session (partial)

In the rest of session 2, Simon has been able to return to the memories of getting into the car, driving to the location of the accident, and seeing the first signs of the accident about to happen (the truck ahead of him starting to lose control). At each step he has experienced discharge of various kinds, including shaking, crying, and angry gestures, each time successfully returning to balance with an increasing sense of well-being and capacity. His phobia of driving has diminished considerably but he still has tension in his arms. Two nights ago he woke from a nightmare drenched in cold sweat.

After an initial greeting and check-in, we begin where we had left off the previous session.

Me: OK Simon, if you feel ready: let's come back again to the moment you first saw the wheels of the truck scoot out sideways. Can you get there?

Simon: Yes, OK, I can see that, a puff of smoke at the wheels and they kick sideways.

Me: (Noticing a slight twisting of his body to the left and a hunching of his shoulders forward) And what else do you notice?

Simon: My shoulders are killing me!

Me: What is that like?

Simon: They're on fire, they feel like they are being twisted off!

Me: And then … what happens now?

Simon: Oh, it's like I have to turn the damn wheel! I can't turn the wheel! I'm going to die!

Me: OK Simon, just feel yourself trying to turn the wheel! Slow it way down! You can give yourself all the time you need, feel what your shoulders are wanting to do!

Simon: (grimaces, groans; very slowly his arms start to move) But I couldn't do it!

Me: But now can you let yourself do what you couldn't do then; give yourself all the time you need…that's it, keep it slow, really feel it—what you couldn't do then, but now you can… that's it, take your time….

Simon: (slowly, with the appearance of a sustained effort, *completes* the gesture of turning the wheel, then slowly relaxes and heaves a huge sigh.) I did it!

Me: What happened, what did you do?

Simon: I turned the wheel even though I was afraid I couldn't. I got out of the way! I went right past, I could see him behind me crashing but I was free!

Me: Great! How does all that power feel?

Simon: It feels fantastic, I feel free, my shoulders feel so light, I don't think I have ever felt like this!

#### Discussion

The SE term for this phenomenon is “*biological completion.*” The ANS and affective subcortical centers are not separate from the somatic, musculoskeletal nervous system. Indeed Panksepp's candidate for the neural substrate of core self (Panksepp, 1998), the PAG, is principally recognized as a nucleus involved in the preparation of instinctive defensive responses. Affective and ANS activation have a direct and immediate effect on the somatic system by way of the EMS (Holstege et al., 1996; Holstege, 2013). Via the reticular formation, the ANS and associated affective and motoric structures change the gamma efferent supply to the muscles, altering the spinal reflexes, muscle tone, and posture in preparation for the movements of fight or flight appropriate to the situation (Bosma and Gellhorn, 1947; Loofbourrow and Gellhorn, 1949; Gellhorn, 1964b). These instinctive affective-motoric (Boadella, 2005) patterned responses have developed to ensure survival; they therefore have an extremely powerful drive to completion. Their organizing nuclei depend partly on proprioceptive feedback from the somatic system to confirm successful completion of the response (Loofbourrow and Gellhorn, 1949; Gellhorn and Hyde, 1953). This is closely related to the phenomena observed by Gellhorn that, absent proprioceptive feedback, the ANS does not reset to baseline (Gellhorn, 1964b). When the survival response is incomplete, ineffective, or prevented, the preparation for the response may persist indefinitely unabated, resulting in continued sympathetic, and in extreme cases concurrent parasympathetic, activation (Gellhorn, 1967b, 1969). This results in a maladaptive organization of the CRN, as the precipitating situation in fact no longer exists. This persistent maladaptation of the CRN is the essence of the stress/trauma state. The organism is no longer actually responding to present conditions, challenging or not, but is locked into an unresolved state of persistent inappropriate activation.

**Figure 7:**
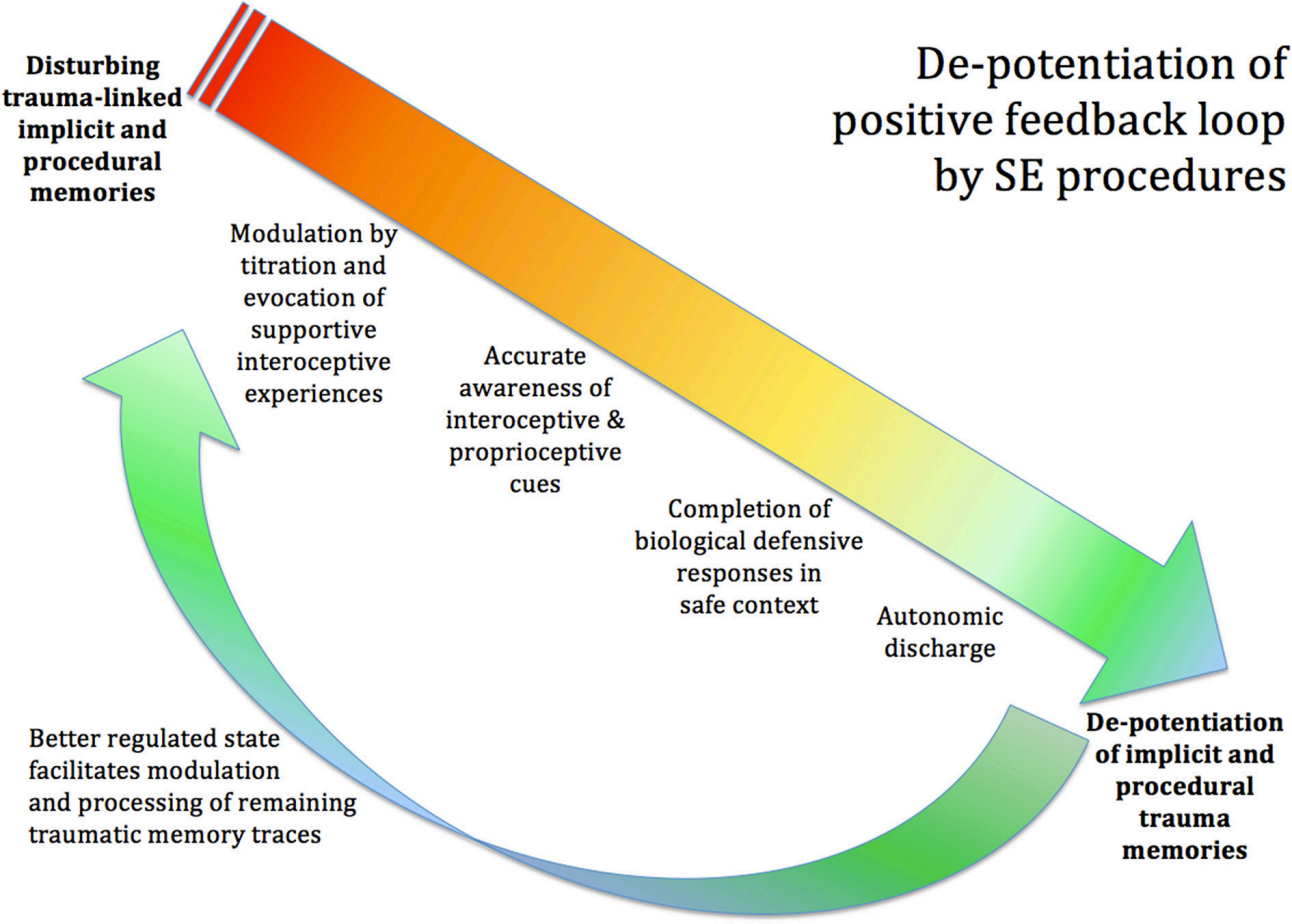
**De-potentiation of positive feedback loop by SE**. The procedures of SE can de-potentiate the disturbing trauma-linked implicit and procedural memories. Titration and the co-evocation of supportive and empowering interoceptive experiences calm the extreme arousal and facilitate accurate awareness of the interoceptive and proprioceptive cues. The client becomes able to identify the urge toward completion of the biological defensive response; and, in the safe and supportive context created by the therapist, is able to complete the blocked defensive response, through imagery and subtle movement. This will often be accompanied by autonomic discharge in the form of heat, trembling, tears, and so on. Once the proprioceptive experience of biological completion has occurred, the memories lose their intense charge, and may now integrate into the hippocampal autobiographical timeline like ordinary memories. Now that the client's nervous system is in a more functional state, the client has more resilience and a greater capacity to tackle any remaining trauma-related memories.

The view of SE is that it is possible to facilitate the completion of this biological defensive response (see Figure 7). This is done through interoceptive and proprioceptive awareness, and may involve imagined “playing out” of a successful resolution of the original (unsuccessful) situation. In other words, this is NOT re-exposure to memory of the original trauma; nor is it a suppression of those memories and feelings. Instead it is a re-working, on a felt subcortical level, which enables the person to have, for the first time, an experience of successful completion of the subcortical instinctive defensive response (Quirin et al., 2011).

The canonical animal model for PTSD is threat coupled with restraint. Restraint alone, without threat, does not induce trauma; nor does threat without restraint (Philbert et al., 2011). The defensive escape response has to be prevented; only then do trauma symptoms develop (Shors et al., 1989). Tellingly, Ledoux found that in rats conditioned through such a procedure to a trauma-like fear response, if they were placed in the same experimental situation and allowed to complete an escape response, the fear conditioning immediately disappeared (Amorapanth et al., 2000).

When the person is finally able to stay fully present to their interoceptive and proprioceptive experience, the interrupted movement (incomplete at the time of the trauma) can then fulfill its meaningful course of action. This gives rise to proprioceptive feedback in the nervous system that tells the ANS that the necessary action has (finally) taken place, so that the sympathetic system can stand down (Gellhorn, 1967b; LeDoux and Gorman, 2001). Careful visual attention, on the part of the therapist, can often detect the interrupted movement behind chronic muscular tension as revealed in very small spontaneous motions; guiding the client to slow things down and take the time they need is essential in order that they can bring these subtle sensations to consciousness. During the precipitating traumatic event, everything happens so fast that they are unable at the time to complete the instinctive survival response; however a fully conscious “replay” of the procedural memory of the event can provide the opportunity for the establishment of a new set of proprioceptive-interoceptive experiences (Mishkin et al., 1984; Redondo, 2014). Sometime just imagining performing the movements brings relief. Studies have shown that imagined movement activates very wide areas of the brain, especially the pre-motor areas which are strongly linked to the autonomic and emotional centers (Decety, 1996; Fadiga et al., 1998; Oishi et al., 2000).

Procedural memory (as distinct from declarative and episodic memory) is the memory of *how to do* things (Squire, 2004), such as riding a bicycle. It is believed to be encoded in the neostriatum rather than the hippocampus (Mishkin et al., 1984), and is not accessible via thoughts or images but via physical sensation (proprioception and kinesthesis) (Mishkin et al., 1984). SE suggests that in a highly stressful situation, vivid procedural memories of the incomplete innate survival actions are laid down, which later intrude and interfere with normal functioning. The intensity of the intrusion is due to the powerful survival imperative embedded in the intrinsically affective content of these defensive reactions; as long as the system does not experience completion, the survival imperative continues to operate, and the person feels *as if* the situation is still happening; this of course is a well-recognized aspect of PTSD. The SE interventions described enable the procedural memories to complete their biological imperative and therefore cease to intrude.

This phenomenon of biological completion is clearly related to that described above as “discharge,” and the necessity for a neuro-muscular (ergotropic) discharge in order to trigger a parasympathetic “reset” (Gellhorn, 1969). This may be a partial explanation for the beneficial effect of vigorous exercise on anxiety and depression (Hötting and Röder, 2013). Our clinical experience seems to indicate, however, that not just any muscular activity will do: profound shifts seem to occur when the activity corresponds to the movement that was interrupted in the precipitating event. I was able to notice subtle hints of the movement (of trying to turn the wheel) manifesting in Simon's body. Once I drew his attention to these, he was able to become aware of the incomplete impulse; the completion of this very specific impulse was crucial in enabling the release of the chronic muscular, autonomic and neuroendocrine activation. It is very unlikely that ordinary voluntary vigorous exercise, even if it had used those same muscles, would have brought about comparable results.

### 4th session (partial)

By now, Simon has completed a lot of work. He has revisited most of the traumatic memories, has experienced considerable autonomic and somatic discharge, and is feeling a great deal better. He sleeps well, is able to concentrate and drives without anxiety. However there is still a mildly “spacy” quality to his presence, and he acknowledges that he does not feel “fully back to myself.” I am aware that we have not yet addressed the actual moment of the accident, which involved violent chaotic motion of the car, out of his control, and the certainty that he was about to die. I suspect the remaining slight dissociation is related to this, and I judge him sufficiently resilient to be able to comfortably handle this last step.

At this point, I ask Simon to recall the first time after the accident at which he really took in that he was OK. He recalled his first interaction with his wife at the hospital, immediately after the accident, recounting a tearful reunion. He had assured his wife that he was fine, exclaiming, “it was a miracle, and I'm OK!” I ask him to notice the feeling in his body as he recalled that scene; he describes a sense of relief, but his expression is a bit flat, without a lot of depth, as if he were recognizing the fact of his survival, but somehow not fully taking it in.

Then I ask him to return to the memory of the moment before the car spun out of control.

Simon: I can feel the steering wheel like iron in my hands—I can see the truck's trailer ahead start to slide sideways—oh God—(I notice his face get pale).

Me: Let's slow down Simon. Feel the chair underneath you…

Simon: (orienting to me a bit) OK….

Me: OK Simon, I'm going to ask you to do something here to help slow things down—it may seem a little strange.

Simon: (still tense, but clearly curious) OK….

Me: We're going to make a sound together, like this: Voooooo (very deep and resonant).

Simon: (smiles a little.) You want me to….

Me: Together now: Vooo….

Simon: (Simultaneously) Voooo..

Me: And again, feel it in your belly: Voooo…

Simon: (noticeably more relaxed) Vooo…

Me: And what do you notice?

Simon: (takes a deep breath) I can feel my legs, my lower body….

Me: What is that like?

Simon: It feels good, solid… I can feel warmth in my legs.

Me: Good, let yourself feel that, take some time… now very gently, touch on that memory again, nice and slow.

Simon: Yes… I can see the trailer ahead…

Me: And what else do you notice?

Simon: I'm gripping the wheel—the lights are so close…

Me: The brake lights?

Simon: Yes… my jaw is so tight, there's nothing I can do, I'm so scared….

Me: Notice your jaw—what is your jaw doing?

Simon: It's shaking, my teeth are chattering.

Me: Ok just let that happen, let your teeth chatter… and what else are you noticing?

Simon: I'm shaking all over, I can't breathe, I feel really scared.

Me: You're doing fine, just let it happen, you are OK, it's your fear and all those pent up tears.

Simon: (shakes and trembles violently, breathes deeply) Oh God, I don't want to die!…. Oh my Lord… I just saw a picture! When I was 7 I fell off my bike, I couldn't breathe. My dad got mad and made me get back on the bike and told me he was proud I didn't cry. I so much wanted to please him, even though I was just a little kid. (Tears start to flow freely down Simon's cheeks as he sobs gently.) I was so scared, so scared…. I think he was scared too; my dad. I think I never really cried after that, not till just now.

Me: You're doing great, let the shaking and tears happen, just feel it… they've been there for such a long time….

(Things settle over a few minutes. Then I notice Simon's body starts to gently jerk in the chair.)

Me: What happens now?

Simon: I'm losing control! It's spinning! The car is spinning.

Me: Slow it down, let's see if you can slow it down like you did before. Feel it, stay with it, it's OK.

Simon: (Gradually his body slows down, comes to rest. He is gently trembling.) I'm alive! I'm alive! (He takes deep spontaneous breaths.)

Me: How does that feel, to be alive?

Simon: (Continuing to sob, though now they appear to be tears of relief and joy.) It's wonderful! I'm alive, I can feel. I thought I was dead, I'm alive! (Gradually the tears subside, his breathing slowly returns to normal, he opens his eyes. He has a quality of intense vitality in his gaze, a softness and aliveness through his body; he looks at me more directly and openly than he has since he started sessions.)

Me: Yes, you are alive. You can feel the joy of being alive through your whole body. Really feel that!

I tell him this is the natural state of his being that becomes available when there are no obstructions. I also explain to him that we all carry many layers of obstruction from past trauma that we may not even remember, that this opening-up is an ongoing process. I suggest that he come in for one more appointment in a month, so we can follow up if there are any remaining issues.

#### Discussion

All the key elements of SE are demonstrated here: presence, embodied resource, titration, pendulation, discharge, and biological completion. Simon is now sufficiently resourced, as a result of the increased resilience of his nervous system gained through the previous work, that he is able to tolerate, befriend and stay fully present to the great fear of dying and the disorienting experiences of being jerked around in the car. The importance of the bodily sensations is clear: the interoceptive experience of shaking and trembling, the kinesthetic/proprioceptive experiences of being jerked around in the car. Titration is evident in the emphasis on slowing down; the use of the “vooo” sound helps generate positive interoceptive sensation to support his capacity to stay present to the extreme fear. We believe that vocalizations like “vooo,” as well as chanting or even song, help to shift the nervous system out of shutdown and then from a sympathetic-dominant to a parasympathetic-dominant state. Mechanisms involved may include (Jerath et al., 2006; Raupach et al., 2008; Chan et al., 2010; Busch et al., 2012; Sano et al., 2014): increased afferent signaling from the diaphragm due to stretching by prolonged exhalation; increased visceral afferent impulses from the abdomen due to sound vibration; and resetting the breathing to a more parasympathetic pattern by lessening CO2 loss by slowing the breath rhythm and extending the exhalation. The deep pitch of the sound may also play a role.

Due to Simon's increased resilience, he does not need nearly as much titration at this stage as he needed at the beginning. He is able to remain present, and to become fully conscious of the events that he had already experienced, but had not been able to “digest” before now.

Not until he has been able to digest the experiences (and experience biological completion) is he able fully to recognize that he has survived. In normal experience, the brain lays down a narrative of life experiences in memory, which can be recalled in sequence and are experienced as belonging to a specific time in the past. This happens in the hippocampus. In parallel, “implicit” memories (Roediger, 1990; Schacter et al., 1993) are laid down in other parts of the brain, including “how-to” memories, probably in the striatum (Reber, 2013), and emotional priming memories in the amygdala (Reber, 2013); there is also evidence that trauma-related memories may be stored in the precuneus and the retrosplenial cortex (Sartory et al., 2013). The trauma-related memories may not form part of a coherent sequential timeline (Van der Kolk and Fisler, 1995), and therefore can be experienced as vivid sensory “flashbacks”: still present, not having receded into the past (Sartory et al., 2013). It has been shown that stress interferes with explicit, autobiographical memory, but not with implicit memory (Luethi et al., 2008); and that stress-related implicit memories can persist indefinitely, even in the absence of conscious recollection of the precipitating situation (Packard et al., 2014). This is believed to be at the root of the pervasive, timeless quality of trauma-related memories (Stolorow, 2003). Only when they have been fully assimilated and assigned to the hippocampal timeline can they become integrated and experienced as “just a memory,” in the past; and only then can one experience oneself as being fully present. In this session, Simon's recovery of the memory of his father making him get back on the bike is pivotal. Although the memory may have been accessible to him prior to the session as a normal autobiographical memory, aspects of the experience (the fear of not being able to breathe, the pushing down of his tears in order to please his father) were encoded as implicit and procedural traumatic memory. The car accident is “layered” on top of the earlier trauma; the bike episode lessened his resilience and impeded his capacity to spontaneously recover from the car accident through emotional, autonomic and motor discharge. The conscious visual *and interoceptive-proprioceptive-kinesthetic* recall of this memory facilitated completion of the interrupted discharge, and enabled a *spontaneous cognitive re-evaluation* of the past event (recognizing his father's fear and the role it played in his actions). Clinical experience in SE shows that such cognitive re-evaluations often emerge *spontaneously* during or shortly after the autonomic and kinesthetic discharges take place. We believe that the subcortical state plays a very significant role in creating and maintaining the faulty cognitive structures, and that cognitive restructuring happens much more easily as the CRN is restored to normal functioning.

## Somatic experiencing: defining the system

When a person is exposed to overwhelming stress, threat or injury, they develop a fixed and maladaptive procedural memory that interferes with the capacity of the nervous system to respond flexibly and appropriately. Trauma occurs when these implicit memories are not neutralized. The failure to restore flexible responsiveness is the basis for many of the dysfunctional and debilitating symptoms of trauma.

In response to threat and injury animals, including humans, execute biologically based, non-conscious action patterns that prepare them to meet the threat by defending themselves. The very structure of trauma, including *activation*, *freezing*, *dissociation*, and *collapse*, is based on the evolution of survival behaviors (Bolles, 1970; Nijenhuis et al., 1998a; Baldwin, 2013). When threatened or injured, all animals draw from a “library” of possible responses. We orient, dodge, duck, stiffen, brace, retract, fight, flee, freeze, collapse, etc. *All* of these coordinated responses are somatically based–they are things that the body does to protect and defend itself.

Animals in the wild recover spontaneously from this state; involuntary movements, changes in breathing patterns, yawning, shaking, and trembling, release or discharge the intense biological arousal; these phenomena have been observed repeatedly by one of the authors (PAL) over 45 years of clinical experience, and confirmed through numerous anecdotal accounts by those who work professionally with wild animals; however we have not been able to find any significant treatment of these phenomena in the peer-reviewed literature. In humans, a variety of factors can thwart this “resetting” of the nervous system: fear of the discharge process itself, prolongation of the traumatic situation, complex cognitive and psycho-social considerations, cortical interference. This failure to reset leaves the nervous system stuck in a dysregulated state. *It is when the spontaneous “reset” fails that we see lasting post-traumatic symptoms*.

The bodies of traumatized people portray “snapshots” of their unsuccessful attempts to defend themselves in the face of threat and injury. Trauma is a highly activated incomplete biological response to threat, *frozen in time*. For example, when we prepare to fight or to flee, muscles throughout our entire body are tensed together in specific patterns of high-energy readiness. When we are unable to complete these appropriate actions, we fail to discharge the tremendous energy generated by our survival preparations. This energy becomes fixed (as a snapshot) in specific patterns of neuromuscular readiness or collapse (i.e., mobilization or immobilization). The person then remains in a state of acute and then chronic arousal and dysfunction in the central nervous system. Traumatized people are not suffering from a disease in the normal sense of the word—they have become stuck in a hyper-aroused or “shutdown” (dissociated) state. It is difficult if not impossible to function normally under these circumstances.

SE avoids asking clients to relive their traumatic experiences, rather it approaches the sensations associated with trauma only after establishing bodily sensations associated with safety and comfort; these become a reservoir of innate, embodied resource to which the individual can return repeatedly as they touch, bit by bit (titration), on the stress-associated sensations. Biological completion and autonomic discharge occur in controlled and manageable steps as the therapist guides the client in attending to visceral sensation or subtle motor impulses associated with incomplete defensive responses.

### Other “bodymind” systems

We believe that the mechanisms elucidated here explain the effectiveness of traditional Asian bodymind systems as well as Western Somatic disciplines and body-oriented psychotherapy. We also believe these mechanisms explain the value of the emphasis on bodily experience, breathing, posture, and balanced muscle tone in seated mindfulness meditation, and extend current theories about the mechanisms behind the long-term beneficial effects of this practice.

In the practice of mindfulness meditation, as well as other forms of contemplative practice, challenging physical and emotional experiences often arise (Kaplan et al., 2012). At times these experiences can pose significant challenges to mental and emotional health, and may lead to the abandonment of the practice. We believe that the SE perspective offers a way of understanding and working with such issues. Although it is beyond the scope of this paper to give an exhaustive treatment, we wish to offer some reflections.

A painful or disturbing interoceptive or proprioceptive experience may be pointing to the necessity for some kind of “biological completion.” Simply maintaining a neutral awareness may not lead to resolution if movement impulses and imagined movements are unconsciously impeded; and many meditation traditions do discourage movement. The question, “what does it feel like my body wants to do?” can often reveal the obstructed impulse, the completion of which may restore comfort and ease.

During contemplative practice, a disturbing experience may arise too intensely or too quickly, resulting in overwhelm and a reactive suppression of the feeling. However, neither overwhelm nor suppression are productive strategies. Temporarily diverting awareness to a positive, safe experience, such as the support of the ground or positive imagery, can allow one to regain inner balance; then a consciously “titrated” process of returning attention to the disturbing experience one *little bit at a time* may facilitate the assimilation of the experience.

The emphasis in mindfulness meditation on remaining detached from discursive thought may sometimes encourage a remote or uninvolved attitude toward arising images, feelings, and insights. We believe that such an attitude may subtly impede the opening-up, de-conditioning process intrinsic to meditation. SE encourages an active, curious exploration of arising phenomena, which is nonetheless not conceptually based. We believe that a familiarity with this form of exploration can inform the practice of mindfulness.

Finally, SE focuses especially on interoceptive and proprioceptive experiences, and puts these in a broad, meaningful framework that can enable one to understand directly the meanings, motivations and implications of such experiences. Traditional Asian practices that emphasize bodily experience, in their full forms, also provide such frameworks (for instance Qigong, Laya Yoga, Tibetan Tsa-Lung practices), but these frameworks may not be appropriate, available, or comprehensible to the Western practitioner. SE provides a broad and sensitive framework firmly rooted in Western scientific understanding, yet also in concert with the above traditional approaches, to help guide one's encounters with difficult material. Moreover it does so without diverting the practitioner into psychological analysis, which may be a significant diversion from the intent of body-focused and meditative practices.

## Summary

While trauma is a nearly ubiquitous human experience, the manifestations of trauma-induced symptoms vary widely. When the nervous system has become “tuned” (Gellhorn, 1967a) by repeated exposure to long-term stress or trauma, the result is manifest in the symptoms of PTSS. Failure to resolve PTSS can evolve into multiple co-morbidities involving the cognitive, affective, immune, endocrine, muscular, and visceral systems. SE is designed to direct the attention of the person to internal sensations that facilitate biological completion of thwarted responses, thus leading to resolution of the trauma response and the creation of new interoceptive experiences of agency and mastery (Parvizi et al., 2013).

### Conflict of interest statement

Peter A. Levine declares that teaching, royalties and consulting related to SE are a source of income. Peter Payne is an SE practitioner (SEP) who derives income from his practice. Mardi Crane-Godreau is an SEP and non-paid member of the Board of Directors of the Somatic Experiencing Trauma Institute™. Somatic Experiencing^®^, SE™, SETI™ and Somatic Experiencing Trauma Institute™ are trademarks owned by Peter A. Levine, or Somatic Experiencing Trauma Institute and are used here with permission from the trademark owners. For more information visit: http://www.traumahealing.org.
