# Corrigendum: Somatic Experiencing: using interoception and proprioception as core elements of trauma therapy

**DOI:** 10.3389/fpsyg.2015.00423

**Published:** 2015-04-14

**Authors:** Peter Payne, Peter A. Levine, Mardi A. Crane-Godreau

**Affiliations:** ^1^Microbiology/Immunology, Geisel Medical School at DartmouthHanover, NH, USA; ^2^Somatic Experiencing Trauma InstituteBoulder, CO, USA

**Keywords:** Somatic Experiencing, body psychotherapy, trauma, interoception, proprioception, core response network

## Introduction

In our recent paper, Somatic Experiencing: Using interoception and proprioception as core elements of trauma therapy (Payne et al., 2015), we stated: “At this point we are not aware of any published peer-reviewed studies of SE, neither case studies, clinical trials, nor tests of its mechanisms.” Unfortunately, we overlooked several papers dealing in whole or in part with Somatic Experiencing® (SE). We wish here to remedy this oversight, with sincere apologies to the authors.

In the peer-reviewed literature, there are two descriptive papers offering brief case studies with commentary on the practice of SE (Levine, 2003; Heller and Heller, 2004); four outcome studies of the use of SE in natural disasters (Leitch, 2007; Parker et al., 2008; Leitch and Miller-Karas, 2009; Leitch et al., 2009); one qualitative study of Gestalt Therapy and SE for back pain (Ellegaard and Pedersen, 2012); one outcome study of military stress resilience training partly based on SE (Stanley et al., 2011) (see also Stanley, 2014); and three hypothesis articles theorizing about aspects of neuroscience pertinent to SE. Two of the latter present conceptual models specifically relevant to SE although they do not focus exclusively on SE (Van der Kolk, 2006; Ruden, 2008); one deals solely with SE (Hricko, 2011). In addition there is one paper not published in a peer-reviewed journal, which addresses ways of measuring the physiological effects of SE (Whitehouse and Poole-Heller, 2009).

## Descriptions of SE

These papers offer case descriptions, with commentary on the principles of SE.

Levine (2003): *Panic, biology, and reason: Giving the body its due*.

Levine's paper discusses the origins of SE, critiques Beck et al.'s (1985) cognitive approach to anxiety disorders, and uses animal behavior as a window on human trauma response. It also presents two detailed case reports.

Heller and Heller (2004): *Somatic Experiencing in the Treatment of Automobile Accident Trauma*.

Heller presents a case study of trauma due to automobile accident, using this as a vehicle to clarify the principles and techniques of SE in a manner similar to our own paper (Payne et al., 2015).

## SE as a trauma intervention in natural disasters

All four papers present a summary of the principles of SE, and make a case for the use of biologically-based interventions as a brief, early intervention for trauma, especially in non-Western cultures. All studies demonstrate significant benefits for the use of SE. All studies discuss the inevitable limitations of studies under field conditions. None of the studies is randomized and fully controlled, but details of the methods are clearly provided. Blinding is largely absent due to its impracticability under these conditions.

Leitch (2007): *Somatic Experiencing Treatment with Tsunami Survivors in Thailand: Broadening the Scope of Early Intervention*.

This paper offers an exploratory study of the use of a brief (1 or 2 sessions) SE-based intervention [Trauma First Aide, developed by Miller-Karas and Leitch (2007), and now called the Trauma Resiliency Model™ (TRM)] with 53 survivors of the 2004 tsunami in Thailand. At 1 year follow-up, 90% of participants reported partial to complete remission of symptoms.

Parker et al. (2008): *Somatic Therapy Treatment Effects with Tsunami Survivors*.

Parker presents a similar study of victims of the same tsunami in southern India. A 75-min SE-based intervention was provided to 150 participants with symptoms of trauma. Several outcome measures were taken at immediate post, 4-week and 8-month follow-up, with significant results indicating substantial benefit. At intake, 80% or participants had one or more PTSD symptoms of arousal and intrusion, and 50% had avoidance symptoms; at 8 months follow-up, 90% had significant or complete improvement.

Leitch et al. (2009): *Somatic Experiencing treatment with Social Service workers following hurricanes Katrina and Rita*.

This paper describes using 1 or 2 sessions of TRM with Social Service workers in the aftermath of hurricanes Katrina and Rita. The treatment group showed significant reduction in PTSD symptoms and increased resilience at 3–4 months follow-up.

Leitch and Miller-Karas (2009): *A case for using biologically-based mental health intervention in post-earthquake China: Evaluation of training in the trauma resiliency model*.

This paper documents the provision of TRM training to 350 disaster responders in Sichuan province, China, after the 2008 earthquake. Ninety seven percent of respondents believed the training would be moderately to very useful in their work.

## SE in military resilience training

Stanley et al. (2011): *Mindfulness-Based Mind Fitness Training: A Case Study of a High-Stress Predeployment Military Cohort*.

Stanley presents an outcome study of Mindfulness-Based Mind Fitness Training (MMFT), derived from SE, TRM, and Mindfulness, with a group of 34 Marine reservists. Increased mindfulness correlated with time spent practicing and with reduced stress.

## SE and gestalt therapy for back pain

Ellegaard and Pedersen (2012): *Stress is Dominant in Patients with Depression and Chronic Low Back Pain*.

Ellegard offers a qualitative study, using a phenomenological-hermeneutic approach, of 6 patients with non-specific low back pain receiving Gestalt Therapy and SE. The study does not enable a separation of the effects of Gestalt Therapy from SE.

## Neuroscience models relevant to SE

Van der Kolk (2006): *Clinical Implications of Neuroscience Research in PTSD;*

Ruden (2008): *Encoding States: A Model for the Origin and Treatment of Complex Psychogenic Pain;*

Hricko (2011): *Whole brain integration in the clinical application of Somatic Experiencing*.

These studies review aspects of neuroscience supportive of the SE approach, and offer conceptual models similar to our own (Payne et al., 2015). Van der Kolk emphasizes evidence supporting the usefulness of attending to interoception and proprioception, and the SE concept of biological completion. Ruden offers hypotheses compatible with SE theory about the neurological mechanisms behind the role of trauma in complex pain. Hricko makes a case for the importance of “right brain literacy” (Hricko, 2011) in SE trauma therapy, referencing research by Schore, Porges and others.

## Physiological measurement in SE

Whitehouse and Poole-Heller (2009): *Heart rate in trauma: Patterns found in Somatic Experiencing and trauma resolution*.

Although it does not appear in a peer-reviewed journal, this paper is nonetheless worthy of mention. It is an informal but suggestive investigation of the use of physiological monitoring to track changes in the nervous system during SE therapy. This is particularly important because SE claims to work primarily via the autonomic nervous system (and other subcortical areas) (Levine, 1977, 2003; Payne et al., 2015). Although some of the measures used may be open to alternate interpretations, his paper offers a valuable methodological perspective. He also presents hypotheses about the correlation of these variables with various stages of SE therapy, and offers examples of measurements taken during SE treatment.

## Summary

Taken together, these papers offer evidence supporting continued research into SE. The papers on disaster response in particular, although not definitive, are strongly suggestive of the efficacy of SE as an early, low-dose, culturally flexible intervention for victims and providers in the context of natural disasters.

### Conflict of interest statement

Peter Payne is an SE practitioner (SEP) who derives income from his practice. Peter A. Levine declares that teaching, royalties and consulting related to SE are a source of income. Mardi A. Crane-Godreau is an SEP and non-paid member of the Board of Directors of the Somatic Experiencing Trauma Institute™.
